# Trace Analysis of Aromatic Compounds in Natural Samples by Shpol’skii Spectroscopy

**DOI:** 10.6028/jres.093.111

**Published:** 1988-06-01

**Authors:** P. Garrigues, E. Parlanti, M. Ewald

**Affiliations:** Groupe d’Océanographie Physico-chimique, UA 348 CNRS Université de Bordeaux I, 33405 Talence Cédex, France

## Introduction

Polycyclic aromatic hydrocarbons (PAH) and their alkylated derivatives are generally mutagenic or carcinogenic compounds and are common trace components of environmental samples. Besides classical analytical methodologies such as capillary gas chromatography and liquid chromatography, high resolution spectrofluorometry (HRS) at low temperature in n-alkane matrices (Shpol’skii effect) is seeing an increasing interest [1–3].

Since the first observation in the early fifties [4], this method has been extensively applied to the analysis of common PAH (e.g., pyrene, benzo(a)pyrene…), of alkylated derivatives [5,6] or heteroatom containing PAH [7,8] in various crude samples or chromatographic extracts. Results presented in this abstract demonstrate the capability of the Shpol’skii spectroscopy to quantify the PAH priority pollutants.

## Experimental Requirements

Fluorescence and phosphorescence spectra of aromatic compounds usually present broad bands at room temperature, having full widths at half-maximum (FWHM) of about 3 nanometers. A sharpening of the luminescence spectra is observed when PAH are incorporated into an appropriate n-alkane matrix frozen at low temperature (*T* ≪ 77 K). The low temperature luminescence spectra exhibit a 0-0 transition with several sharp peaks (“quasi-lines”) having FWHM about 0.1 nm called multiplet structure and related to different substitution sites of the aromatic molecules in the n-alkane lattice [9].

Experimental conditions for obtaining sharp emission spectra are specific. Particularly, a remarkable matching in length of long axis and short axis of the guest (aromatic) and the host (n-alkane) molecules seems to exist [10]. When this “key and hole” rule is not respected, broad band or complicated quasilinear emission spectra are observed.

A preliminary fast freezing of solution at 77 K in liquid nitrogen is also necessary to avoid aggregate formation [11]. Another feature is also the concentration dependence of the intensity of the quasilines. However at low concentration (about 10^−6^ M and less), the formation of aggregates is minimized and the reproducibility of fluorescence intensity is not altered [11,12].

## Quantitative Analysis of Selected PAH

**Figure 1:**
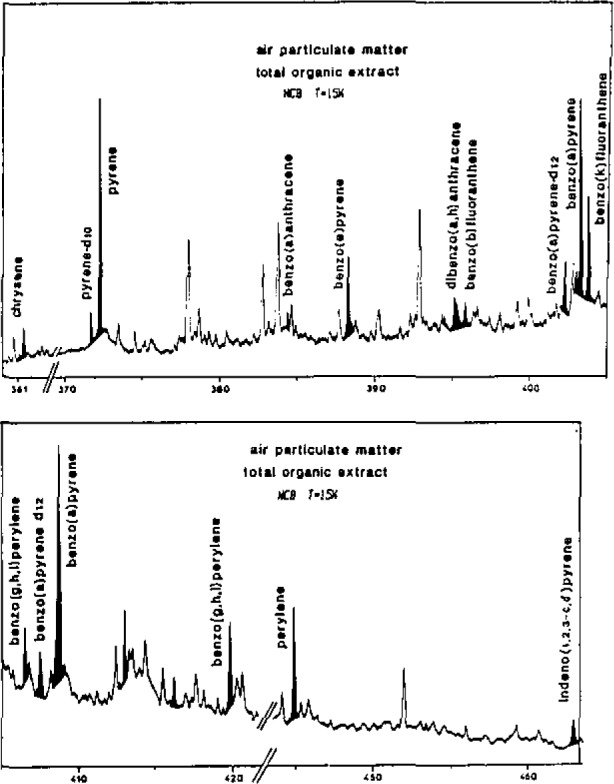
Fluorescence emission spectra of the organic air particulate extract in n-octane at 15 K. Deuterated pyrene and benzo(a)pyrene have been added as internal standards. Excitation wavelengths centered respectively at 275 nm (top) and at 290 nm (bottom).

The American Environmental Protection Agency (EPA) has retained about 16 PAH as priority pollutants [13]. They have been partially identified by GC-MS or HPLC. Most of these molecules exhibit well resolved emission spectra (fig. 1) in n-octane at 15 K by using a low temperature spectrofluorometer previously described [14]. Perdeuterated PAH (deuterated pyrene and benzo(a)pyrene) have been used as internal standards and have been added to the total organic extract. These compounds exhibit a similar Shpol’skii emission spectra to that of the respective parent compound but with a spectral line shift of about 1 nm.

**Figure 2:**
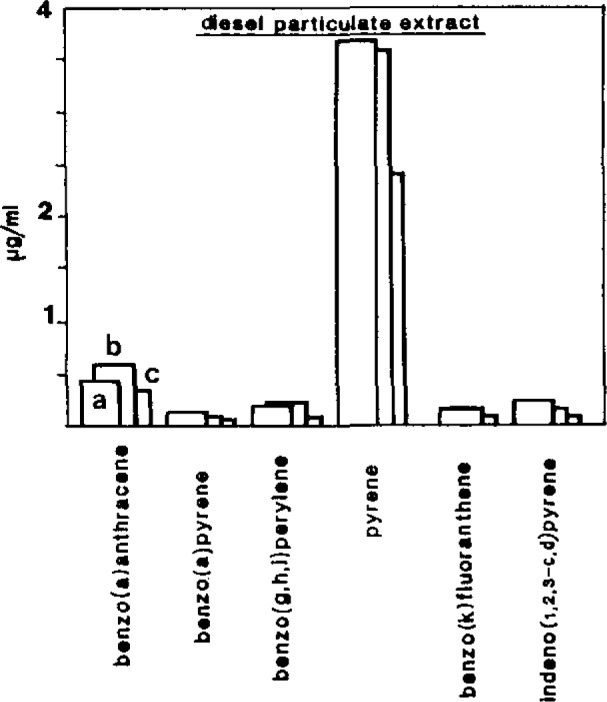
Comparison of the concentration values obtained on a diesel particulate extract for PAH determined by several analytical techniques (International Round Robin organized by the National Bureau of Standards, Gaithersburg, MD, USA): a) Capillary gas chromatography coupled to mass spectrometry (NBS values, ref. 15). b) Liquid chromatography coupled to spectrofluorometry (ref. 15). c) Shpol’skii spectroscopy values.

Most of the EPA PAH could easily be identified and quantified by this technique. Low molecular weight aromatic compounds (acenaphthene, ace-naphthalene, fluorene, phenanthrene and anthracene) were difficult to detect due to their low fluorescence quantum yield. To evaluate the accuracy of this method, we have analyzed several organic extracts (diesel particulate, air particulate) used for intercalibration exercises organized by the National Bureau of Standards (Gaithersburg, MD USA). The results (imprecision less than 15%) obtained by Shpol’skii spectroscopy have been compared with values obtained by other analytical methodologies (HPLC coupled with fluorescence detection, GC coupled to mass spectrometry). As presented in figure 2, good agreement is observed among the three analytical techniques, demonstrating the reliability of Shpol’skii spectroscopy for quantitative analysis.

## Conclusion

Partial results presented here show the analytical capability of high resolution spectrofluorometry in Shpol’skii matrices for the determination of different priority pollutant aromatic compounds in the total organic extract of natural samples.

## References

[1] Colmsjö A, Östman CE (1980). Anal Chem.

[2] D’Silva AP, Fassel VA (1984). Anal Chem.

[3] Garrigues P, Ewald M (1983). Anal Chem.

[4] Shpol’skii EV (1962). Sov Phys Usp (Eng Transi).

[5] Garrigues P, Ewald M (1983). Org Geochem.

[6] Garrigues P, Parlanti E, Radke M, Willsch H, Ewald M (1987). J Chromatog.

[7] Garrigues P, De Vazelhes Ewald M, Joussot-Dubien J, Schmitter JM, Guiochon G (1983). Anal Chem.

[8] Dorbon M, Schmitter JM, Garrigues P, Arpino P, Ewald M, Guiochon G (1984). Org Geochem.

[9] Lamotte M, Merle AM, Joussot-Dubien J, Dupuy F (1975). Chem Phys Lett.

[10] Garrigues P, De Sury R, Bellocq J, Ewald M (1985). Analysis.

[11] Garrigues P, Lamotte M, Ewald M, Joussot-Dubien J (1981). C R Acad Sci série II.

[12] Garrigues P (1985). Thèse de doctorat ès Sciences.

[13] (1977). US-EPA Effluents Guidelines Division.

[14] Soclo HH, Garrigues P, Ewald M (1986). Analysis.

[b15-jresv93n3p441_a1b] 15May, W. E., Personal Communication (1987).

